# Worldwide malaria incidence and cancer mortality are inversely associated

**DOI:** 10.1186/s13027-017-0117-x

**Published:** 2017-02-14

**Authors:** Li Qin, Changzhong Chen, Lili Chen, Ran Xue, Ming Ou-Yang, Chengzhi Zhou, Siting Zhao, Zhengxiang He, Yu Xia, Jianxing He, Pinghua Liu, Nanshan Zhong, Xiaoping Chen

**Affiliations:** 1 State Key Laboratory of Respiratory Disease, Guangzhou Institutes of Biomedicine and Health, Chinese Academy of Sciences, 190 Kaiyuan Avenue, Guangzhou Science Park, 510530 Guangzhou, China; 2 State Key Laboratory of Respiratory Disease, Guangzhou Institute of Respiratory Disease, First Affiliated Hospital, Guangzhou Medical University, 151 Yanjiang Road, 510120 Guangzhou, China; 3 Channing Laboratory, Brigham and Women’s Hospital, 181 Longwood Ave, Boston, MA 02115 USA; 4 Boston University, Boston, MA 02215 USA; 5 Department of Bioengineering, McGill University, Montreal, QC H3A 0C3 Canada

**Keywords:** Malaria incidence, Cancer mortality, Generalized additive mixed model (GAMM), Epidemiological data, Regression analysis

## Abstract

**Background:**

Investigations on the effects of malaria infection on cancer mortality are limited except for the incidence of Burkitt’s lymphoma (BL) in African children. Our previous murine lung cancer model study demonstrated that malaria infection significantly inhibited tumor growth and prolonged the life span of tumor-bearing mice. This study aims to assess the possible associations between malaria incidence and human cancer mortality.

**Methods:**

We compiled data on worldwide malaria incidence and age-standardized mortality related to 30 types of cancer in 56 countries for the period 1955–2008, and analyzed their longitudinal correlations by a generalized additive mixed model (GAMM), adjusted for a nonlinear year effect and potential confounders such as country’s income levels, life expectancies and geographical locations.

**Results:**

Malaria incidence was negatively correlated with all-cause cancer mortality, yielding regression coefficients (log scale) of −0.020 (95%CI: −0.027,-0.014) for men (*P* < 0.001) and-0.020 (95%CI: −0.025,-0.014) for women (*P* < 0.001). Among the 29 individual types of cancer studied, malaria incidence was negatively correlated with colorectum and anus (men and women), colon (men and women), lung (men), stomach (men), and breast (women) cancer.

**Conclusions:**

Our analysis revealed a possible inverse association between malaria incidence and the mortalities of all-cause and some types of solid cancers, which is opposite to the known effect of malaria on the pathogenesis of Burkitt’s lymphoma. Activation of the whole immune system, inhibition of tumor angiogenesis by *Plasmodium* infection may partially explain why endemic malaria might reduce cancer mortality at the population level.

**Electronic supplementary material:**

The online version of this article (doi:10.1186/s13027-017-0117-x) contains supplementary material, which is available to authorized users.

## Background

Some parasites have been implicated as risk factors for certain cancers, including *Schistosoma haematobium* for bladder cancer and *Clonorchis sinensis* for liver cancer and bile duct cancer [1]. However, other parasites, such as *Trypanosoma cruzi, Toxoplasma gondii, Toxocara Canis*, and *Acanthamoeba castellani,* have been suggested to improve the survival of cancer-bearing mice [2]. Regarding malaria parasites specifically, there are two opposing viewpoints. In 1981, Greentree proposed that malaria infection might enhance the host’s immune system and could serve as an adjuvant to conventional cancer therapy [3]. In contrast, in 1990, Eze suggested that malaria infection might trigger the production of reactive oxygen species or could activate oncogenes and eventually lead to cancer [4]. One previous study suggested that malaria incidence and all-cancer mortality might be positively correlated [5]. The author compared malaria incidence data from 1994 with all-cancer mortality for the period 1950–1994 in United States. However the results were limited because the time spans for cancer mortality and malaria incidence did not match, and there were many potential confounders, particularly due to variations in economic development and immigration across the states. In 1994, the entire United States had fewer than 1000 imported cases of malaria, and it is known that immigrants usually target more economically advanced cities. It has been known that a positive correlation exists between malaria infection and the incidence of Burkitt’s lymphoma (BL) in African children [6, 7]. However, no study has systematically examined the correlation between endemic malaria and the incidences or mortality rates of other cancers.

Some reports using animal models have suggested a link between malaria infection and the incidences or mortality rates of certain cancers. Trager et al. reported that the plasma of malaria-infected chickens could inhibit a chicken Rous I tumor [8]. Contradictory results have been reported for liver cancer and malaria in rat models [9, 10]. We recently demonstrated that malaria infection inhibited tumor growth and metastasis and prolonged the survival of tumor-bearing mice in a murine Lewis lung cancer (LLC) model [11]. *Plasmodium yoelii* 17XNL (a benign form of a murine malaria parasite) infection inhibited tumor angiogenesis in mice and induced innate and adaptive antitumor responses in LLC-bearing mice, leading to the induction of antitumor and anti-metastatic activities.

To examine whether such an association also exists in humans, we systematically analyzed malaria and all-cause cancer and 29 individual cancer statistical data in 56 countries from WHO publications and databases for the period 1955–2008.

## Methods

### Malaria data

Data regarding malaria cases for the following time periods were collected from the following sources: 1955–1964, WHO Epidemiological and Vital Statistics Report (1966); 1962–1981, WHO World Health Statistics Annual (1983); 1982–1997, WHO Weekly Epidemiological Record (1999); and 1990–2008, WHO 2009 annual report. In the event of a short overlap between two reports, data from the later report were used. We compiled worldwide malaria data from 218 countries for the period 1955–2008. Worldwide population data for 228 countries during the period 1955–2008 were obtained from the International Database of the U.S. Census Bureau (http://www.census.gov). The malaria incidence was calculated by dividing the number of reported malaria cases by the country’s overall population; malaria incidence data were produced for 170 countries in this way.

### Cancer data

Age-standardized mortality data on all-cause cancer and 29 individual types of cancer (1955–2008) from 194 countries were obtained from the WHO cancer mortality database (http://www-dep.iarc.fr/WHOdb/WHOdb.htm); the database provides statistics separately for each gender. As shown in Additional file 1: Table S1, merging the cancer mortality data (1955–2008) with the malaria incidence data provided combined data for 56 countries. Income information for these 56 countries was obtained from the World Bank database (http://data.worldbank.org/country). Data on life expectancies at birth (both sexes combined) and location data from the 56 countries were obtained from a publication of the Population Division of the United Nations Department of Economic and Social Affairs (World Population Prospects: The 2012 Revision; http://esa.un.org/wpp/).

### Epidemiological data analysis

Using longitudinal malaria incidence and cancer mortality data from 56 countries, we examined trends in malaria incidence and all-cause cancer mortality for each country via linear regression. We then applied a generalized additive mixed model (GAMM) to examine the association between malaria incidence and cancer mortality. GAMM has the ability to set a random intercept to fit a wide range of cancer mortality rates among countries, to examine the nonlinear relationship between malaria incidence and cancer mortality, and to adjust for a nonlinear year effect and potential confounders such as country’s income levels, life expectancies and geographical locations.

We first examined the distributions of the malaria incidence and cancer mortality rates and then log-transformed the malaria incidence and cancer mortality rates before inputting the rates into the models. All the analyses were performed using Empower software (www.empowerstats.com, X&Y solutions, Inc., Boston, MA) and R software (http://www.R-project.org).

## Results

Of the 56 countries examined, the data integrity was different from country to country. The 56 countries provide average 23-year data, while Venezuela has 50-year data, but both Barbados and Kuwait have only 6-year data. Twenty nine countries provide the data of 1955–1981, 9 countries provide the data of 1982–2008, 18 countries have the data of 1955–2008. Thirty four countries locate in Asia/Africa/Latin America, among them 3 (Egypt, Mauritius and South Africa) in Africa, and 22 in Eruope/North America/Oceania. Twenty five countries were classified as high-income (high), and 31 were classified as low- and middle-income (low).

We first drew a smooth curve fitting the malaria incidence and the all-cause cancer mortality. The year 1981 was defined as a turning point; in this year, the malaria incidence increased, and the all-cause cancer mortality decreased significantly. We thus divided the study period into two periods: 1955–1981 and 1982–2008 in the following analysis. In addition, 24 countries showed a significant (*P* <0.05) increasing trend (up) in malaria incidence, 8 countries showed a significant decreasing trend (down) in malaria incidence, and 24 countries showed a non-significant change over the years (no change). We summarized the malaria incidence and all-cause cancer mortality data for the 56 countries from 1955–2008 in Table 1.

**Table 1:** Characteristics of malaria incidence and all-cause cancer mortality in 56 countries

Country	Period	N	Malaria incidence		All cancers			
					Male		Female	
			Median (Min-Max)	Yr. ratio^a^	Median (Min-Max)	Yr. ratio	Median (Min-Max)	Yr. ratio
Argentina	1966-2008	43	1.80 (0.26-7.08)	0.97	152.62 (132.01-199.59)	0.99	95.59 (86.39-124.56)	0.99
Armenia	1982-2008	22	4.84 (0–37.91)	1.24*	135.54 (115.84-150.75)	1.01	80.115 (69.29-90.5)	1.01
Australia	1955-1981	27	1.29 (0.39-4.30)	1.07*	151.21 (128.66-164.78)	1.01	98.43 (95.23-101.73)	1.00
Austria	1955-1981	26	0.08 (0.01-1.24)	1.15*	189.84 (181.83-199.04)	1.00	128.935 (113.45-138.06)	0.99
Azerbaijan	1982-2007	22	4.7 (0.28-161.40)	1.1	116.3 (77.16-149.99)	0.98	64.825 (47.8-79.77)	0.99
Barbados	1976-1981	6	0.60 (0.40-2.82)	0.75	125.56 (118.13-141.62)	1.01	102.3 (90.87-107)	0.98
Belgium	1955-1981	21	0.05 (0.01-0.60)	1.14*	195.63 (153.64-216.35)	1.01	117.84 (111.95-126.6)	1.00
Belize	1971-1999	21	1505.52 (26.4-4696.7)	1.15*	58.13 (26.39-147.75)	1.02	55.69 (36.51-131.28)	1.01
Brazil	1979-2008	28	281.77 (124.94-404.38)	1.01	93.62 (85.36-103.3)	1.01	67.41 (63.96-72.99001)	1.00
Bulgaria	1964-1981	17	0.15 (0.05-4.74)	1.28*	128.08 (117.99-142.31)	0.99	81.82 (77.03-89.66)	0.99
Canada	1955-1981	24	0.03 (0–2.57)	1.22*	150.21 (134.13-162.86)	1.01	109.23 (104.19-115.47)	1.00
Colombia	1955-2008	39	301.58 (81.42-588.20)	1.01	96.9 (61.78-103.77)	1.01	85.15 (71.38-97.2)	1.00
Costa Rica	1961-2008	47	34.45 (4.54-279.67)	1	103.62 (87.74-124.21)	1.00	88.79 (65.97-100.72)	1.00
Cuba	1970-1981	12	1.29 (0.01-5.90)	1.61*	133.21 (123.91-139.03)	0.99	92.24 (85.03-95.82)	0.99
Denmark	1955-1981	26	0.33 (0.11-2.15)	1.09*	165.54 (151.26-181.88)	1.00	135.72 (125.75-145.28)	1.00
Dominican Republic	1970-2005	31	14.05 (3.15-83.90)	1	42.58 (34.04-61.54)	1.01	39.33 (34.97-51.95)	1.00
Ecuador	1977-2008	29	362.59 (34.07-890.54)	0.98	71.42 (55.09-78.69)	1.00	71.52 (59.06-78.98)	1.00
Egypt	1955-2008	17	0.10 (0.01-474.70)	0.85*	41.35 (22.02-50.13)	1.01	26.23 (13.32-33.14)	1.02
El Salvador	1958-2008	34	147.79 (0.55-1924.15)	0.88*	39.99 (21.8-60.4)	1.02	54.05 (34.52-68.83)	1.01
Finland	1965-1981	15	0.09 (0.02-0.47)	1.16*	184.27 (177.38-195.1)	0.99	98.47 (94.04-106.99)	0.99
France	1955-1981	24	0.04 (0–1.03)	1.14*	185.97 (149.46-207.7)	1.01	100.655 (94.08-106.6)	0.99
Georgia	1990-2007	16	0.43 (0.02-9.25)	1.43*	79.99 (62.51-102.93)	1.00	52.14 (41.88-66.36)	1.00
Greece	1961-1981	21	0.55 (0.30-1.77)	0.96*	125.41 (116.45-145.84)	1.01	75.95 (71.89-82.94)	1.01
Guatemala	1963-2008	25	445.36 (69.81-1004.5)	1.01	67.03 (38.95-78.97)	1.02	75.595 (50.07-84.01)	1.01
Hungary	1955-1981	27	0.06 (0.01-0.46)	0.98	170.12 (130.85-214.48)	1.02	121.71 (114.18-129.34)	1.00
Ireland	1955-1981	17	0.07 (0.03-0.98)	1.13*	158.37 (128.77-169.4)	1.01	121.11 (107.84-130.69)	1.01
Israel	1975-1981	7	0.48 (0.27-1.16)	1.24*	131.36 (118.82-137.7)	0.99	113.88 (107.84-116.67)	0.99
Italy	1955-1981	27	0.07 (0.01-0.43)	1.11*	160.04 (123.09-187.98)	1.02	100.25 (96.73-103.36)	1.00
Japan	1955-1981	27	0.03 (0.01-0.07)	1.03	143.55 (125.39-149.85)	1.00	94.36 (84.6-99.17)	0.99
Kuwait	1975-1981	6	11.93 (7.15-16.04)	0.88*	94.08 (89.14-142.41)	0.95	73.56 (56.31-85.91)	1.02
Kyrgyzstan	1990-2008	19	0.27 (0–54.73)	1.34	103.2 (95.9-139.63)	0.98	66.97 (62.79-79.04)	1.00
Mauritius	1961-1989	29	5.57 (1.26-67.34)	1.08	96.93 (72.36-134.73)	1.01	68.86 (55.38-84.72)	0.99
Mexico	1969-2008	32	22.03 (2.14-174.16)	0.9*	73.52 (51.76-76.71)	1.01	68.87 (64.13-73.67)	1.00
New Zealand	1955-1981	26	0.48 (0.09-2.23)	1.04	157.77 (134.46-175.78)	1.01	114.795 (108.98-124.02)	1.00
Nicaragua	1973-2008	25	524.44 (13.95-1679.01)	0.94	49.55 (15.79-59.14)	1.04	52.13 (21.9-61.56)	1.03
Norway	1955-1981	21	0.08 (0.03-1.39)	1.15*	134.65 (124.81-145.52)	1.01	101.08 (97.48-110.9)	1.00
Panama	1955-2008	47	53.18 (5.81-670.11)	0.96*	73.97 (57.86-91.1)	1.01	66 (55.64-81.41)	1.00
Paraguay	1988-2008	15	21.94 (6.34-187.26)	0.94	72.11 (66.2-74.86)	1.00	63.07 (58.71-65.54)	1.00
Peru	1966-2007	30	179.07 (16.11-984.90)	1.08*	63.9 (40.08-74.8)	1.00	65.13 (38.9-77.01)	1.00
Philippines	1988-2008	15	54.54 (24.62-249.58)	0.91*	92.9 (78.82-100.9)	1.01	65.715 (57.07-71.5)	1.01
Poland	1959-1981	22	0.04 (0.01-0.10)	1.04*	153.43 (103.15-181.65)	1.02	102.625 (81.88-106.09)	1.01
Portugal	1955-1981	27	1.38 (0.04-10.35)	1.05	121.39 (89.41-133.25)	1.01	84.4 (75.34-90.29)	1.00
Romania	1963-1981	13	0.08 (0.02-0.10)	1.03	127.47 (124.17-131.21)	1.00	86.39 (83.02-98.06)	0.99
Singapore	1963-1981	19	11.53 (7.05-22.80)	0.98	171.88 (157.07-207.55)	1.01	100.32 (91.53-116.34)	1.01
South Africa	1988-2008	17	30.16 (13.08-143.40)	0.97	136.65 (121.25-145.78)	0.99	84.31 (70.13-87.88)	1.01
Spain	1955-1981	27	0.09 (0.02-8.57)	0.94*	134.17 (105.68-151.66)	1.01	86.23 (79.24001-91.09)	1.00
Suriname	1971-2008	26	2058.43 (152.56-14273.09)	1.12*	69.16 (34.15-95.24)	1.00	65.26 (26.55-95.75)	0.99
Sweden	1955-1981	27	0.30 (0.05-1.48)	1.14*	131.41 (117.91-148.03)	1.01	109.24 (101.89-113.42)	1.00
Switzerland	1955-1981	22	0.12 (0.02-2.15)	1.19*	174.17 (169.64-183.12)	1.00	111.08 (102.52-123.75)	0.99
Tajikistan	1982-2005	22	75.25 (3.32-4508.57)	1.46*	71.17 (52.63-110.44)	0.97	52.985 (36.33-69.2)	0.98
Thailand	1959-2006	27	175.6 (41.84-843.59)	0.99	58.68 (13.05-89.11)	1.04	36.42 (10.44-56.02)	1.04
Trinidad and Tobago	1955-1981	8	20.54 (0.27-213.59)	0.8	93.44 (83.49-116.19)	1.00	106.055 (88.44-123.58)	0.99
Turkmenistan	1990-1998	9	0.29 (0.03-3.21)	1.39	102.3 (87.32-132.92)	0.95	66.835 (55.55-81.32)	0.96
United Kingdom	1962-1981	20	0.54 (0–3.65)	1.41*	186.43 (180.55-189.06)	1.00	120.165 (114.59-123.92)	1.00
Uruguay	1963-1978	8	0.04 (0.03-0.15)	0.95	200.58 (192.12-205.04)	1.00	126.605 (119.13-133.04)	0.99
Venezuela	1955-2007	50	66.1 (7.5-249.34)	1.04*	98.74 (89.01-113.05)	1.00	94.185 (77.43-141.76)	0.99

Notes: ^a^Yr. ratio: annual change ratio calculated as 10^β, where β is the regression coefficient of the annual log-transformed malaria incidence or cancer mortalities. *P < 0.05

Spline smoothing by the GAMM showed a linear inverse relationship between malaria incidence and all-cause cancer mortality. A further analysis using the linear term of malaria incidence showed that a 10-fold increase in malaria incidence was associated with a lower log-transformed all-cause cancer mortality (−0.31 [95% CI:-0.37,-0.25], *P* <0.001, for men and −0.32 [95% CI:-0.37,-0.26], *P* <0.001, for women; Table 2). After adjusting for year, life expectancy at birth, income level, and geographical locations, this negative relationship persisted (regression coefficients: −0.20 [95% CI:-0.27,-0.14], *P* <0.001, for men and −0.20 [95% CI:-0.25,-0.14], *P* <0.001, for women). A stratified analysis showed an inverse relationship between malaria incidence and all-cause cancer mortality regardless of time periods, national income levels, geographical locations, or the malaria incidence trend (Table 2).

**Table 2:** Regression analysis of the relationship between all-cancer mortality and malaria incidence, 1955–2008

Model	Male	Female
Basic model	−0.31 (−0.37, −0.25) ***	−0.32 (−0.37, −0.26) ***
Adjusted model	−0.20 (−0.27, −0.14) ***	−0.20 (−0.26, −0.14) ***
Period		
1955-1981	−0.11 (−0.16, −0.05) ***	−0.05 (−0.10, 0.00) **
1982-2008	−0.24 (−0.34, −0.15) ***	−0.11 (−0.19, −0.03) **
Income		
Low	−0.28 (−0.37, −0.18) ***	−0.25 (−0.34, −0.16) ***
High	−0.11 (−0.15, −0.06) ***	−0.07 (−0.11, −0.03) ***
Geographical location		
Asia/Africa/Latin America	−0.27 (−0.36, −0.18) ***	−0.24 (−0.33, −0.16) ***
Europe/North America/Oceania	−0.16 (−0.20, −0.12) ***	−0.10 (−0.13, −0.06) ***
Malaria incidence trend		
Down	−0.20 (−0.31, −0.08) ***	−0.25 (−0.36, −0.13) ***
No change	−0.13 (−0.26, 0.00) *	−0.06 (−0.18, 0.06)
Up	−0.21 (−0.32, −0.10) ***	−0.03 (−0.12, 0.07)

Notes

1. Both malaria incidence and cancer mortality data were log transformed. The results shown are 10 times the regression coefficients and 95% confidence intervals

2. The basic model was adjusted for year; the adjusted model was adjusted for year, life expectancy at birth, national income level and geographical location

3. ***P <0.001; **P <0.01; *P <0.05

We then analyzed 29 individual types of cancer, and found that the inverse relationship between malaria incidence and cancer mortality was significant for colorectum and anus (both men and women), colon (both men and women), lung (men), stomach (men) and breast (women) cancer (Table 3). For other individual cancers, the correlation was not as significant as for these cancers. Detailed analyses of other cancer types are given in Additional file 2: Table S2.

**Table 3:** Regression analysis of the relationship between mortality rates associated with 5 individual types of cancer and malaria incidence, 1955–2008

Cancer	Basic model	Adjusted model	Period	
			1955-1981	1982-2008
Colon				
Male	−0.72 (−0.87, −0.56) ***	−0.52 (−0.68, −0.36) ***	−0.31 (−0.48, −0.14) ***	−0.59 (−0.81, −0.38) ***
Female	−0.87 (−1.02, −0.72) ***	−0.63 (−0.78, −0.48) ***	−0.39 (−0.54, −0.24) ***	−0.64 (−0.86, −0.43) ***
Colon, rectum and anus				
Male	−0.51 (−0.62, −0.41) ***	−0.37 (−0.47, −0.26) ***	−0.25 (−0.37, −0.13) ***	−0.41 (−0.58, −0.25) ***
Female	−0.58 (−0.68, −0.48) ***	−0.42 (−0.52, −0.32) ***	−0.34 (−0.46, −0.22) ***	−0.45 (−0.60, −0.29) ***
Lung				
Male	−0.36 (−0.46, −0.25) ***	−0.20 (−0.32, −0.09) ***	−0.24 (−0.35, −0.12) ***	−0.54 (−0.71, −0.37) ***
Breast				
Female	−0.46 (−0.56, −0.37) ***	−0.30 (−0.40, −0.20) ***	−0.20 (−0.29, −0.10) ***	−0.32 (−0.45, −0.19) ***
Stomach				
Male	−0.66 (−0.76, −0.55) ***	−0.39 (−0.50, −0.28) ***	−0.31 (−0.40, −0.21) ***	−0.23 (−0.37, −0.08) ***

Notes

1. Both malaria incidence and cancer mortality data were log transformed. The results are 10 times the regression coefficients and the 95% confidence intervals

2. The basic model was adjusted for year; the adjusted model was adjusted for year, life expectancy at birth, national income level and geographical location

3. ***P <0.001

The epidemiological analysis suggested that an inverse relationship exists between malaria incidence and mortality due to colorectum and anus, colon, lung, breast and stomach cancer; however, no significant correlations were found between malaria incidence and other cancers.

## Discussion

In the present study, we utilized WHO cancer and malaria data to conduct longitudinal (1955–2008) analyses to assess the relationship between these diseases. Our analyses indicate that endemic or epidemic malaria may decrease mortality for some solid cancers, including colon cancer both in men and women, lung and stomach cancer in men, and breast cancer in women. Country-specific cancer incidence and mortality are associated with many factors, such as ethnicity, habits, customs, the level of economic development, the level of health care, and the level of cancer diagnosis and treatment. The geographical distribution of the countries is also a potential confounding factor. Malaria is widely transmitted in Africa, Asia, and Latin America but has a low incidence in industrialized countries. Another potential confounding factor is the effect of time. To reduce the effects of these confounding factors, a GAMM was used to analyze the longitudinal data. A random intercept effect for each country in the GAMM regression analysis was used to accommodate the variations in cancer mortality across different countries. After adjusting for year, life expectancy at birth, national income level, and geographical locations, the inverse relationship between malaria incidence and all-cause cancer mortality persisted. Recent studies have demonstrated that some antimalarial drugs, such as artemisinin and chloroquine, have antitumor activities [12, 13]; thus, malaria treatment in endemic areas may affect cancer mortality, as well. However, although artemisinin was only used in clinics after the 1990s [14, 15], our analysis indicated that the negative relationship between malaria incidence and all-cause cancer mortality existed before the 1990s, during the period 1955–1981. Thus, the clinical use of artemisinin should not be a major confounder. Chloroquine may have been widely used throughout the entire period 1955–2008, but it was used only for short-term (3-day) treatment of malaria; thus, it is unlikely to have significantly affected cancer mortality. Furthermore, our murine Lewis lung cancer (LLC) model studies have demonstrated that 3-day treatment with a clinically relevant dose of chloroquine does not improve the survival of tumor-bearing mice (data not shown). Therefore, it is less likely that the observed inverse relationship between malaria incidence and cancer mortality was caused by the confounding factors discussed above.

Increased levels of economic development and increased life span typically decrease malaria incidence and increase cancer mortality. Thus, in countries with low levels of malaria and controlled endemic malaria, an inverse relationship between malaria incidence and cancer mortality would be observed merely through a simple analysis. To address this issue, we included a year variable spline smoothing term in the model to control for this time effect. We also conducted simulation analyses to estimate class I errors and the statistical power using data from 8 countries with decreasing malaria incidence. The estimated probability of this finding being due to chance was <0.035, and the power to detect our observed effect (−0.03) was 0.752. We also stratified the countries by trends in malaria incidence (up, down or no change). Negative correlations between malaria incidence and cancer mortality were observed in each stratum. Another potential time effect was the changing population structure over time because aging that relates to cancer mortality may also relate to malaria incidence. To address this issue, we stratified the years into two segments, 1955–1981 and 1982–2008, to narrow the aging effect. Negative correlations between malaria incidence and cancer mortality were still observed during each time period.

There are three limitations to the present study. First, cancer mortality and malaria incidence data were obtained from WHO publications and databases. Different countries may use different reporting criteria for the malaria incidence data. In addition, we were unable to obtain age-standardized malaria incidence data from existing databases. For cancer mortality, although the data were age-standardized for the countries studied, medical care and cancer reporting systems varied across countries and may have changed over time; these factors may affect the comparability of cancer mortality data between countries and years. Although our analysis using the GAMM approach maximally accommodated the limitations of existing data by primarily examining trends over time within each country instead of across countries, such limitations could not be completely eliminated by our data analysis. Second, we do not have other countries’ data, especially the data from malaria high endemic countries in Africa. Therefore the impact of endemic malaria on cancer mortality in our analysis may mainly reflect *vivax* malaria that prevails outside Africa, not *falcipuram* malaria that prevails in Africa, even though all four species of human malaria parasite (*Plasmodium vivax, falcipuram, ovale and malariae*) contain pathogen-associated molecular patterns (PAMPs [16]) that can trigger the antitumor immune response (see below). Third, the positive relationship between the incidence of Burkitt’s lymphoma in African children and *falciparum* malaria has been well established, because this malaria causes a prolonged expansion of B cell in the germinal centers and therefore provides more time for DNA damage and MYC oncogene translocation that ultimately leads to Burkitts lymphoma [17], but we were unable to obtain mortality or incidence data for this tumor from WHO publications or databases. Thus, our analysis model lacks information that could be used to establish a positive relationship between malaria incidence and cancer mortality; such information would be important for validating our model further.

Malaria infection may serve to enhance immune surveillance mechanisms against some types of solid cancers. During the course of malaria, *Plasmodium* PAMPs [16] as danger signals are detected by the host immune cells’ sensors called pattern recognition receptors (PRRs) which include the toll-like receptors (TLRs) [18] at the membrane of endosomes or on the cell surface, RIG-I-like receptors (RLRs) [19] and NOD-like receptors (NLRs) [20] localized in cytoplasm. The *Plasmodium* PAMPs include the known glycosylphosphatidylinositol anchors (GPI anchors) [21], haemozoin [22] and immunostimulatory nucleic acid motifs [23] and other unknown molecules [24]. The PRRs activated by *Plasmodium* PAMPs trigger distinct transcriptional programs and stimulate multiple downstream pathways to induce systemic immune responses [25], including release of pro-inflammatory and Th1-type cytokines such as TNF-α, IL-1β, IL-2, IL-6, IL-12, type I and type II IFNs [25, 26], activation of NK cells, NKT cells, γ/δ T cells, macrophages and dendritic cells (DCs), afterwards activation of CD4+ and CD8+ T cells [26, 27] that counteract the tumor immune-suppressive microenvironment that contains TGF-β, IL-10, regulatory T cells (Tregs) and myeloid-derived suppressive cells (MDSCs) [28, 29], then turn the immune-suppressive milieu to immune-supportive milieu, finally may transform the tumor into an effective tumor vaccine [30, 31]. On the other hand, malaria damage-associate molecular patterns (DAMPs), such as the known intrinsic uric acid, microvesicles and haem [31–33] also induce similar immune activity. Indeed, our previous study demonstrated that blood-stage malaria exhibits anti-tumor effects by inducing a potent anti-tumor innate immune response, including the secretion of IFN-γ and TNF-α and the activation of NK cells. Our murine lung cancer model studies also demonstrated that malaria infection induced adaptive anti-tumor immunity by increasing tumor-specific T-cell proliferation and the cytolytic activity of CD8^+^ T cells and increased the infiltration of these cells into tumor tissues. In these studies, we found that in approximately 10% of lung cancer (LLC)-bearing mice infected with malaria parasite, the tumor regressed and did not regrow when the mice were re-inoculated with the same cancer cells, most likely because of the long-term memory of specific antitumor cellular immunity [11]. Study by Deng XF and colleagues demonstrated that attenuated liver-stage *Plasmodium* inoculation induced antitumor innate immune response, including secretion of TNF-α, IL-6/12 and IFN-γ and antitumor adaptive immunity with increasing cytolytic activity of CD8+ T cells [34]. Our unpublished data suggest that blood-stage *Plasmodium* infection significantly decreases the numbers of MDSCs in breast cancer (4 T1)-bearing mice or Tregs in lung cancer (LLC)-bearing mice. In addition, our previous study indicated that malaria infection significantly inhibited tumor angiogenesis in mice [11]. A review by Hobohm [35] suggested that the fever induced by malaria infection may cause an increase in tumor cell death. Based on our previous study and our unpublished data, parasitemia is required to effectively inhibit tumor growth. However, in mice, *Plasmodium* infection causes only a short-term infection without fever. Repeated *Plasmodium* infection is difficult to observe in murine models [36]. In humans lacking effective antimalarial treatment, *Plasmodium* infection can cause long-term parasitemia that is accompanied by a high fever in the acute phase, and this syndrome can recur many times throughout the life span [37]. Therefore, a naturally acquired *Plasmodium* infection by mosquito bite would produce liver-stage and blood-stage malaria that sequentially stimulate the immune systems to turn the tumor into the effective tumor vaccine, in combination with fever during the acute phase and the inhibition of tumor angiogenesis. In medical literature, febrile infection was linked to spontaneous regression of tumor [38], and malaria is a typical febrile infection. Other pathogen infections may have similar impact on cancer mortality or morbidity [39, 40], due to a similar mechanism of PAMPs-triggered antitumor immunity [41], but malaria parasite may be more significant than other pathogens, because malaria contains two stages (liver and blood) of infection, manifests typical high fever paroxysms during acute phase and may last longer period if not treated with effective antimalarial drugs. In addition, malaria is a well-documentary infectious disease in WHO databases and publications partially due to easily to be diagnosed by fever paroxysm and a simple blood smear test. Other pathogen infections lack well-documentary data in WHO databases or publications for analysis. In summary, some or all of these above mentioned factors and mechanisms may explain why endemic malaria might reduce cancer mortality at the population level.

## Conclusion

We report an inverse association between malaria incidence and the mortality rates of all-cause and several types of solid cancers. However, because this study is a descriptive retrospective analysis, we are unable to draw a causal relationship between malaria and the mortality rates of some cancers. More well-defined prospective epidemiological studies are required to confirm this relationship. We recommend that public health officials in the WHO and in individual countries coordinate malaria control programs and cancer epidemiological studies in the future. In addition, more comprehensive mechanistic studies should be conducted.

## Additional files

Additional file 1: Table S1. Combined data of cancer mortality data (1955–2008) with the malaria incidence data for 56 countries. (XLS 1007 kb)
Additional file 2: Table S2. Regression analysis of Malaria incidence and other individual cancer mortality. (XLS 55 kb)

## Abbreviations

BL: Burkitt’s lymphoma
CI: Coefficience interval
CSA: Chondroitine Sulfate A.
DCs: Dendritic cells
GAMM: Generalized additive mixed model
GPI: Glycosylphosphatidylinositol anchors
LLC: Lewis lung cancer
MDSCs: Myeloid-derived suppressive cells
NLRs: NOD-like receptors
PAMPs: Pathogen-associated molecular patterns
Pf: Plasmodium falciparum
PRRs: Pattern recognition receptors
RLRs: RIG-I-like receptors
TLRs: Toll-like receptors
Tregs: regulatory T cells
WHO: World Health Organization

## References

[1] de Martel C, Ferlay J, Franceschi S, Vignat J, Bray F, Forman D, Plummer M (2012). Global burden of cancers attributable to infections in 2008. A review and synthetic analysis. Lancet Oncol.

[2] Darani HY, Yousefi M (2012). Parasites and cancers: parasite antigens as possible targets for cancer immunotherapy. Future Oncol.

[3] Greentree LB (1981). Malariotherapy and cancer. Med Hypotheses.

[4] Eze MO, Hunting DJ, Ogan AU (1990). Reactive oxygen production against malaria--a potential cancer risk factor. Med Hypotheses.

[5] Lehrer S (2010). Association between malaria incidence and all cancer mortality in fifty U.S. States and the district of Columbia. Anticancer Res.

[6] Whittle HC, Brown J, Marsh K, Greenwood BM, Seidelin P, Tighe H (1984). T-cell control of Epstein-Barr virus-infected B cells is lost during P. Falciparum malaria. Nature.

[7] Rochford R, Cannon MJ, Moormann AM (2005). Endemic Burkitt’s lymphoma: a polymicrobial disease?. Nat Rev Microbiol.

[8] Trager W, McGhee RB (1953). Inhibition of chicken tumor I by plasma from chickens infected with an avian malaria parasite. Proc Soc Exp Biol Med.

[9] Angsubhakorn S, Bhamarapravati N, Sahaphong S, Sathiropas P (1988). Reducing effects of rodent malaria on hepatic carcinogenesis induced by dietary aflatoxin B1. Int J Cancer.

[10] Angsubhakorn S, Sathiropas P, Bhamarapravati N (1986). Enhancing effects of rodent malaria on aflatoxin B1-induced hepatic neoplasia. J Cancer Res Clin Oncol.

[11] Chen L, He Z, Qin L, Li Q, Shi X, Zhao S, et al. Antitumor effect of malaria parasite infection in a murine lewis lung cancer model through induction of innate and adaptive immunity. PLoS One. 2011;6(9). 10.1371/journal.pone.0024407 PMC3170332 21931708

[12] Kimura T, Takabatake Y, Takahashi A, Isaka Y (2013). Chloroquine in cancer therapy: a double-edged sword of autophagy. Cancer Res.

[13] Tilaoui M, Mouse HA, Jaafari A, Zyad A (2014). Differential effect of artemisinin against cancer cell lines. Nat prod bioprospecting.

[14] White NJ (1996). The treatment of malaria. N Engl J Med.

[15] Karbwang J, Na-Bangchang K, Thanavibul A, Bunnag D, Chongsuphajaisiddhi T, Harinasuta T (1994). Comparison of oral artesunate and quinine plus tetracycline in acute uncomplicated falciparum malaria. Bull World Health Organ.

[16] Takeuchi O, Akira S (2010). Pattern recognition receptors and inflammation. Cell.

[17] Robbiani DF, Deroubaix S, Feldhahn N, Oliveira TY, Callen E, Wang Q (2015). *Plasmodium* infection promotes genomic instability and AID-dependent B cell lymphoma. Cell.

[18] Schroder K, Tschopp J (2010). The inflammasomes. Cell.

[19] O’Neill LA, Golenbock D, Bowie AG (2013). The history of toll-like receptors - redefining innate immunity. Nat Rev Immunol.

[20] Barbalat R, Ewald SE, Mouchess ML, Barton GM (2011). Nucleic acid recognition by the innate immune system. Annu Rev Immunol.

[21] Durai P, Govindaraj RG, Choi S (2013). Structure and dynamic behavior of toll-like receptor 2 subfamily triggered by malarial glycosylphosphatidylinositols of plasmodium falciparum. FEBS J.

[22] Kalantari P, DeOliveira RB, Chan J, Corbett Y, Rathinam V, Stutz A (2014). Dual engagement of the NLRP3 and AIM2 inflammasomes by plasmodium-derived hemozoin and DNA during malaria. Cell Rep.

[23] Sharma S, DeOliveira RB, Kalantari P, Parroche P, Goutagny N, Jiang Z (2011). Innate immune recognition of an AT-rich stem-loop DNA motif in the plasmodium falciparum genome. Immunity.

[24] Liehl P, Zuzarte-Luis V, Chan J, Zillinger T, Baptista F, Carapau D (2014). Host-cell sensors for plasmodium activate innate immunity against liver-stage infection. Nat Med.

[25] Gazzinelli RT, Kalantari P, Fitzgerald KA, Golenbock DT (2014). Innate sensing of malaria parasites. Nat Rev Immunol.

[26] Punsawad C (2013). Effect of malaria components on blood mononuclear cells involved in immune response. Asia Pac j Trop Biomed.

[27] Inoue S, Niikura M, Mineo S, Kobayashi F (2013). Roles of IFN-gamma and gammadelta T cells in protective immunity against blood-stage malaria. Front Immunol.

[28] Smyth MJ, Ngiow SF, Ribas A, Teng MW (2016). Combination cancer immunotherapies tailored to the tumour microenvironment. Nat Rev Clin Oncol.

[29] van der Burg SH, Arens R, Ossendorp F, van Hall T, Melief CJ (2016). Vaccines for established cancer: overcoming the challenges posed by immune evasion. Nat Rev Cancer.

[30] van den Boorn JG, Hartmann G (2013). Turning tumors into vaccines: co-opting the innate immune system. Immunity.

[31] Figueiredo RT, Fernandez PL, Mourao-Sa DS, Porto BN, Dutra FF, Alves LS (2007). Characterization of heme as activator of toll-like receptor 4. J Biol Chem.

[32] Mantel PY, Marti M (2014). The role of extracellular vesicles in plasmodium and other protozoan parasites. Cell Microbiol.

[33] Gallego-Delgado J, Ty M, Orengo JM, van de Hoef D, Rodriguez A (2014). A surprising role for uric acid: the inflammatory malaria response. Curr Rheumatol Rep.

[34] Deng X, Dong Z, Quanxing L, Yan D, Xu W, Qian C (2016). Antitumor effect of intravenous immunization with malaria genetically attenuated sporozoites through induction of innate and adaptive immunity. Int J Clin Exp Pathol.

[35] Hobohm U (2001). Fever and cancer in perspective. Cancer Immunol Immunother: CII.

[36] Dascombe MJ, SJIMA (1994). The absence of fever in rat malaria is associated with increased turnover of 5-hydroxytryptamine in the brain. Temperature regulation: advances in pharmacological sciences.

[37] Fernando SD, Gunawardena DM, Bandara MR, De Silva D, Carter R, Mendis KN (2003). The impact of repeated malaria attacks on the school performance of children. AmJTrop Med Hyg.

[38] Hoption Cann SA, van Netten JP, van Netten C (2003). Dr william Coley and tumour regression. A place in history or in the future. Postgrad Med J.

[39] Mastrangelo G, Fadda E, Milan G (1998). Cancer increased after a reduction of infections in the first half of this century in Italy: etiologic and preventive implications. Eur J Epidemiol.

[40] Keef R, Jonas WB, Knogler W, Stenzinger W. Fever, cancer incidence and spontaneous remissions. Neuroimmunomodulation. 2011;9(2):55-64. 10.1159/000049008 11549887

[41] Kucerova P, Cervinkova M. Spontaneous regression of tumour and the role of microbial infection--possibilities for cancer treatment. Anticancer Drugs. 2016;27(4):269–77. 10.1097/CAD.0000000000000337 PMC4777220 26813865

